# Comparative Analysis of Tumor‐Immune Microenvironment Diversity Among Biopsy, Resection, and Metastatic Colorectal Cancer Specimens

**DOI:** 10.1155/jimr/9281559

**Published:** 2026-04-25

**Authors:** Qi Liu, Wei Liu, Chunmei Zhang, Yixuan Gao, Dongmei Feng, Duo Deng, Yun Pan

**Affiliations:** ^1^ Dali University School of Basic Medicine, Dali, Yunnan, China; ^2^ Shenzhen Hospital, Guangzhou University of Traditional Chinese Medicine, Shenzhen, Guangzhou, China, gzucm.edu.cn; ^3^ The First Affiliated Hospital of Dali University, Dali, Yunnan, China, dali.edu.cn

**Keywords:** CD4+T/CD8+T cell infiltration, colorectal cancer, microsatellite instability, PD-L1, tumor-immune microenvironment

## Abstract

**Background:**

Colorectal cancer (CRC) tumor immune microenvironment (TIME) components such as programmed death‐ligand 1 (PD‐L1) and CD4+T/CD8+T cells play an important role in immunotherapy, which is closely related to the treatment and prognosis of patients. Due to the fact that CRC is relatively insidious and most of it has advanced and metastasis when discovered, it is important to use biopsy specimens to accurately assess the TIME before treatment. However, there are no studies investigating the association of TIME in biopsy and excision specimens and in metastatic colorectal lesions.

**Methods:**

This study compares PD‐L1 expression and CD4+T and CD8+T cell infiltration as representative indicators of CRC TIME among endoscopic biopsy specimens (*n* = 20), surgical resection specimens (*n* = 20), metastasis specimens (*n* = 29), microsatellite instability (MSI) specimens (*n* = 15), and microsatellite stable (MSS) specimens (*n* = 78).

**Results:**

(1) There was a positive correlation of PD‐L1 in tumor cells (TCs) from biopsy and resection (*p* < 0.05) but not in interstitial cells (ICs) (*p* > 0.05). CD4+T and CD8+T cells were positively correlated in biopsy and resection specimens (*p* < 0.05). (2) There are no significant differences in PD‐L1–positive cells and CD4+T/CD8+T cell infiltrations between primary lesions of nonmetastatic patients and primary lesions of metastatic patients. (3) Compared with MSS CRC, MSI CRC had higher PD‐L1 expression, CD4+T/CD8+T cells, and Ki67‐positive rates (*p* < 0.05).

**Conclusion:**

CD4+T/CD8+T cell infiltration and PD‐L1 expression in resected specimens can be used to predict the progression and growth environment of patients’ tumors to a certain extent, which is convenient for clinicians to predict treatment and medication in advance. Metastatic CRC has significant differences in tumor infiltration and MSI from nonmetastatic CRC.

## 1. Introduction

Colorectal cancer (CRC) has been of great concern to the World Health Organization (WHO) due to its high global disease burden, with its mortality rate ranking second and incidence rate third among all common malignancies worldwide [1, 2]. It has been reported that there were ~500,000 new CRC cases worldwide by 2022, and disparities in its morbidity and mortality exist across different countries and regions, which are attributed to variations in medical care access and dietary habits [3]. Specifically, the incidence of CRC is considerably higher in Europe and North America, while it is relatively lower in Africa, Central Asia, and South Asia [4]. According to statistics from the American Cancer Society, 53,000 deaths from CRC were recorded in the United States in 2023. Since 2000, the overall incidence and mortality of CRC in the United States have exhibited a declining trend; however, the proportion of CRC cases occurring in individuals under 55 years of age has risen from 11% in 1995 to 20% in 2019 [5]. According to relevant surveillance data, by 2022, there were 517,100 new CRC cases in China, accounting for ~10.7% of all malignant tumors nationwide. In the same year, 240,000 deaths from CRC were reported in China, with the national age‐standardized incidence and mortality rates standing at 36.63 and 17.00 per 100,000 population, respectively; both indices have shown an overall increasing trend in recent years [6].

It is well established that colonoscopy combined with subsequent endoscopic resection of colorectal neoplasms reduces the morbidity and mortality of CRC, and this beneficial effect is predominantly observed in highly developed countries. Although surgical resection of colorectal tumors has become the mainstay of treatment for CRC, 30%–50% of patients still develop recurrence or metastasis following curative‐intent therapy, with the 5‐year overall survival rate plummeting to just 10% in these patients [7]. According to recent statistics, the overall survival rate of patients with metastatic CRC remained below 15% as of 2023 [5]. Therefore, it is critical to reduce the metastatic potential of CRC and improve the efficacy of therapeutic interventions for this disease.

During tumorigenesis and subsequent metastasis, malignant cells undergo progressive diversification and heightened heterogeneity, leading to tumor infiltration by a multitude of immune‐related components—including distinct cytokine/chemokine milieus, cytotoxic activity, and immunosuppressive factors. Such immune heterogeneity is ubiquitous across nearly all solid tumors and exhibits spatiotemporal changes with tumor progression and therapeutic interventions, and it is closely associated with disease advancement and treatment response, particularly in the field of immunotherapy. Thus, an accurate understanding of tumor immune heterogeneity is pivotal for the development of effective therapeutic strategies. Aided by multiregion and omics sequencing, single‐cell sequencing, and longitudinal liquid biopsy approaches, recent studies have revealed the complexity of investigating tumor immune heterogeneity and its potential clinical relevance for immunotherapy. Elucidating the mechanisms underlying the heterogeneity of the tumor immune microenvironment (TIME) will facilitate the clinical assessment of tumor heterogeneity and further drive the development of more effective personalized therapies.

One factor contributing to the heterogeneity of CRC is the TIME, which is dynamic and complex, comprising diverse immune cells, cytokines, and associated proteins, acts as the primary effector site for adaptive immune responses and exerts a pivotal role in tumorigenesis, tumor progression, and therapeutic response [8]. TIME is influenced by multiple factors. For example, in terms of tumor location, right‐sided colon tumors exhibit higher lymphocyte infiltration compared to left‐sided colon tumors [9]. Regarding age, although the overall infiltration of immune cells in the tumor microenvironment of young patients with metastatic CRC is similar to that of older patients, their functional status is more active [10]. In terms of biopsy depth, the number of CD8+T cells is higher at the invasive margin, but their functional impairment is also more significant compared to the tumor center [10, 11]. As for treatment modality, patients with CRC who receive neoadjuvant therapy show a significant reduction in CD8+ cytotoxic T cells in the tumor microenvironment, presenting a state of “immune hyperresponsiveness” [12]. TIME is complex and heterogeneous, and the density and diversity of tumor‐infiltrating immune cells (tumor‐infiltrating lymphocytes [TILs]), including CD4+T cells and CD8+T cells, are closely associated with treatment efficacy and patient prognosis. Single‐cell analyses have revealed that CD4+T cells are constitutive residents of the TIME in multiple tumor lesions [13–17]. The role of CD4+T cells in the TIME has been identified as an essential component of antitumor immunity. RNA sequencing has confirmed that CD4+T cells mediate antitumor immunity [18, 19] and have the ability to predict the efficacy of antiprogramed death 1 (PD‐1) therapy in bladder cancer [20, 21]. CD8+T cells exert a more potent immune effect; upon activation, they differentiate into CD8+ cytotoxic T lymphocytes (CTLs) and exert cytotoxic effects by infiltrating tumor cells (TCs). Reversing CD8+T cell exhaustion is a major antitumor mechanism of immune checkpoint inhibitors (ICIs), and this parameter can also predict the efficacy of ICI therapy in hepatocellular carcinoma (HCC) [22]. PD‐1 (CD279) is a type I transmembrane protein composed of 288 amino acid residues and a member of the CD28 family. It is widely expressed on a variety of immune cells, such as activated T cells, B cells, NK cells, and DC cells, and programmed death‐ligand 1 (PD‐L1) (CD274) serves as its ligand. Upon interaction with PD‐L1, PD‐1 is activated and recruits the phosphatase SHP‐2 to the T cell receptor (TCR) and the costimulatory molecule CD28. This process leads to decreased phosphorylation of key components in the TCR and CD28 signaling pathways, thereby reducing their activity. Consequently, this mechanism suppresses T cell proliferation, reduces cytokine secretion, alters cellular metabolic processes, impairs the function of CTLs, and ultimately induces apoptosis of activated T cells. The PD‐1/PD‐L1 pathway governs the induction and maintenance of immune tolerance in the tumor microenvironment and plays a pivotal role in tumor immune evasion. Since the first evidence demonstrating an association between PD‐L1 protein expression and the efficacy of anti‐PD‐1 checkpoint blockade therapy was reported, PD‐L1 expression levels have been adopted as a companion diagnostic to predict clinical responses to ICI immunotherapy in various solid tumors. However, PD‐L1 expression exhibits remarkable heterogeneity across intratumoral, intertumoral, spatial, and temporal dimensions; for example, after evaluating PD‐L1 expression in primary tumors and brain metastases from NSCLC patients, a significant difference in PD‐L1 expression between the two types of lesions was observed, which impairs the efficacy of immunotherapy in patients with solid tumors.

Immunohistochemical quantification of DNA mismatch repair (MMR) proteins enables the classification of CRC into MMR‐deficient (dMMR) or MMR‐proficient (pMMR) subtypes [23]. Microsatellite instability (MSI) is predominantly caused by functional defects in the MMR system resulting from the loss of MMR protein expression. Therefore, detecting the absence of these proteins can serve as an indirect indicator of MSI status. This subtype accounts for ~15% of all CRC cases and only 5% of metastatic CRC patients. It is characterized by a high tumor mutation burden (TMB) [24], resulting in low sensitivity to conventional chemotherapy, poor prognosis, and high responsiveness to immunotherapy. Neoadjuvant therapy may confer clinical benefits to patients with locally advanced CRC (LACRC); however, it is not suitable for patients with dMMR or MSI status. Investigating microsatellite status differences between primary and metastatic CRC patients, as well as comparing the similarities and discrepancies in immune‐related biomarkers between these two cohorts, may provide valuable insights for identifying treatment strategies for metastatic CRC.

In summary, during tumor progression, nearly all patients with solid tumors will experience alterations in the TIME and develop resistance to immunotherapy. The coevolution of tumors and immune compartments sustains the heterogeneity of the immune microenvironment, particularly under the pressure of therapeutic interventions. Assessments of T cell infiltration or PD‐L1 expression in tumor tissue samples hold significant potential for guiding personalized immunotherapy, thereby addressing the challenges posed by immune microenvironment heterogeneity.

Therefore, this study will compare the TIME components (PD‐L1, CD4+T cells, and CD8+T cells) in surgical specimens, endoscopic biopsy specimens, and metastatic specimens; investigate their similarities and discrepancies; and further evaluate the feasibility of using endoscopic biopsy specimens to predict the TIME status of surgical specimens.

## 2. Methods

### 2.1. Patients and Specimens

We performed a comprehensive analysis of patients with preserved:

(1) Paraffin‐embedded tissue samples and corresponding clinical information of patients diagnosed with CRC who underwent surgical resection; 85 qualified CRC specimens were selected as the CRC group.

(2) Endoscopic biopsy specimens and corresponding surgical resection specimens (including primary tumor and adjacent peritumoral tissues) were rigorously matched to ensure that each biopsy sample originated from the same lesion as its corresponding resection specimen. All specimens obtained from patients who had undergone prior radiotherapy or chemotherapy were excluded from the study. The tumor cellularity in each biopsy specimen was evaluated using a combination of gross and microscopic examination, and only specimens with ≥50% TC content were included to avoid interference from normal tissue contamination. The biopsy tissue fragments are measured between 0.1 and 0.4 mm^3^ on average. All biopsy procedures were performed by experienced endoscopists under high‐definition endoscopy, with the biopsy depth strictly controlled at 2–3 mm (penetrating through the muscularis mucosa into the submucosa). This depth was confirmed by pathological examination to ensure adequate sampling of both tumor tissue and the adjacent microenvironment for subsequent analyses.

(3) Immunohistochemistry (IHC) was performed to evaluate the expression of four core MMR proteins (MLH1, MSH2, MSH6, and PMS2) in tumor tissues. The patient’s own normal tissue (such as adjacent non‐neoplastic mucosa or intratumoral lymphocytes) served as a positive internal control for protein expression. Interpretation followed established criteria: Loss of nuclear expression of any one of the four proteins in the TCs was defined as dMMR. Intact nuclear expression of all four proteins in TCs was defined as pMMR. Multiplex fluorescence PCR was employed to amplify five mononucleotide repeat microsatellite markers (BAT25, BAT26, MONO‐27, NR‐21, and NR‐24). Capillary electrophoresis was performed using an ABI 3500 Genetic Analyzer, and fragment sizes were analyzed with GeneMapper software. Interpretation was conducted by comparing the electrophoretic profiles of tumor tissue DNA with matched normal tissue DNA from the same patient: If new allelic fragments (i.e., shifts) were observed at two or more loci, the sample was classified as MSI‐high (MSI‐H); if instability was detected at only one locus, it was classified as MSI‐low (MSI‐L); and if no instability was observed at any locus, it was classified as microsatellite stable (MSS). In this study, MSI‐H status is referred to as MSI, while MSS and MSI‐L statuses were combined into an MSS group.

(4) Tissue samples of metastatic lesions (to qualify, patients must have metastatic disease detected in traditional imaging studies) with CRC peritumoral tissues as the control group, and 29 qualified cases with both primary CRC samples and distant metastatic samples were selected as the CRC metastasis group. All cases were reviewed by at least two senior pathologists. The diagnosis is independently completed by pathology experts, the different opinions are discussed collectively, and the final results are subsequently given.

### 2.2. IHC

Formalin‐fixed paraffin‐embedded (FFPE) tissue blocks of colorectal adenocarcinoma derived from endoscopic biopsies and surgical resections were cut into 4‐μm‐thick sections, which were dewaxed in xylene and sequentially hydrated in a graded ethanol series. Antigen retrieval was then performed via microwave irradiation in 0.5 mM EDTA buffer (pH 8.0) for 20 min. After blocking with normal goat serum, the sections were incubated overnight at 4°C with the corresponding primary antibodies: anti‐PD‐L1 (CST, cat. no. 13684 s), anti‐CD4 (Maixin, cat. no. RMA‐0620, ready‐to‐use), and anti‐CD8 (Maixin, cat. no. MAB‐0021, ready‐to‐use). Subsequently, the sections were rinsed three times with phosphate‐buffered saline (PBS). Finally, the sections were incubated with the peroxidase/diaminobenzidine (DAB) Rabbit/Mouse secondary antibody from the EnVision detection system (Dako/Agilent Technologies, cat. no. K5007, ready‐to‐use) for 30 min at room temperature (RT). DAB chromogen from the aforementioned secondary antibody staining kit was added for color development, followed by counterstaining with hematoxylin for 2 min at RT. Human lymph node or tonsil tissues were used as positive controls, and negative controls were established by replacing the primary antibody with isotype‐matched nonimmune immunoglobulins.

### 2.3. Assessment of PD‐L1, CD4+T/CD8+T Cell Expression, and the Infiltration Level of Infiltrated Immune Cells

Immunohistochemical staining results were independently evaluated by two pathologists who were blinded to the clinicopathological data of patients. In cases where the counting discrepancy was within 5%, a re‐evaluation was performed to reach a consensus. The enumeration of CD4+T and CD8+T cells was conducted under a light microscope (Olympus BX‐43): 10 high‐power fields (HPFs) with abundant positive cells were selected at 400× magnification (0.2375 mm^2^), the number of immune cells in each field was recorded, and the mean value was calculated to determine the average number of positive cells per 400× HPF (0.2375 mm^2^). The positive staining pattern of CD4 and CD8 molecules was characterized by cytoplasmic immunoreactivity with enhanced membranous staining. For each sample, the CD4+/CD8+T cell ratio was calculated by dividing the average count of CD4+T cells by that of CD8+T cells. Only TILs within the borders of invasive tumors (within 0.5 mm of the tumor parenchyma) and TILs in the center of the tumor and tumor surface were included in the enumeration; ulcerated areas, ulcer bases, necrotic regions, and areas with high‐grade intraepithelial neoplasia were excluded from the assessment [25, 26].

PD‐1 expression in TILs was evaluated, and the average number of PD‐1 positive cells was assessed on 10 HPFs (Olympus BX‐43, 400X, 0.2375 mm^2^). We separately calculated the proportion of PD‐L1 expression on TCs and within the tumor stroma [27–29]. Expression of PD‐L1 in TCs was considered positive when membrane staining was present in more than 1% of TCs, while for PD‐L1 expression in TILs, either membrane or cytoplasmic staining in more than 1% of TILs was considered positive [30].

In addition, all PD‐L1–positive immune cells associated with adenomas, dysplasia, ulcers, chronic gastritis, or any other processes not directly attributable to the tumor were explicitly excluded from the scoring.

### 2.4. Bioinformatics Analysis

We searched and analyzed the correlation between PD‐L1, CD8, and CD4 genes in CRC through the GEPIA2 database (http://gepia2.cancer-pku.cn/#index), and if the *p*‐value was less than 0.05, it could be considered correlated of these two genes in CRC. TIMER 2.0 database (http://timer.comp-genomics.org/) [31–33] and TISIDB database were used to analyze the relationship between PD‐L1 and the immune infiltration of CD4+T/CD8+T cells in the TIME of CRC, and when the *p*‐value was less than 0.05, it was considered to be correlated.

### 2.5. Statistical Analysis

The experimental results and corresponding clinical data were processed using GraphPad Prism V.9 (La Jolla, California, USA) or SPSS V.26.0 (Chicago, Illinois, USA). The experimental data of CD4+T/CD8+T cells were first processed to eliminate extreme values, and subsequent normality testing confirmed that all data met the requirements of a normal distribution for parametric statistical analysis. To assess the correlations between PD‐L1 and CD4/CD8, Spearman’s correlation analysis was used. This choice was made because PD‐L1 expression was dichotomized (high vs. low) based on the median, rendering it a noncontinuous variable that violates the prerequisites for Pearson’s correlation. CD4 and CD8 correlation was performed using Pearson’s correlation analysis. A two‐sided *t* test was used, and *p*  < 0.05 was considered statistically significant. For non‐normally distributed paired comparisons of PD‐L1 expression between endoscopic biopsy and resection specimens, the Wilcoxon matched‐pairs signed‐rank test was used. Statistical power analysis for the biopsy/resection group, MSI/MSS groups, and control/metastasis groups was performed using R software (version 4.3.1). For multiple comparisons across panels, *p*‐values were adjusted using the Benjamini–Hochberg false discovery rate (FDR) correction. An FDR‐adjusted *p*‐value < 0.05 was considered statistically significant.

## 3. Results

### 3.1. Clinicopathological Characteristics of Endoscopic Biopsy and Resection Specimens

Sufficiently matched endoscopic biopsy and resection specimens (including primary and peritumoral tissues) from 20 CRC patients in 2020 were identified. We also conducted a detailed statistical analysis of the patients’ pathological data (ethnicity, survival status, tumor size, TNM stage, number of chemotherapy treatments and medications, tumor markers, and preoperative and postoperative NLR).

Specimens and pathological information of 20 patients, including eight colon cancer and 10 rectal cancer patients, were included (male: female: 8:12), with 62.0 for median age (range 27–86). Most patients were nonsmokers (85%), and only one patient had a history of alcohol consumption (5%). The mean size of the tumor sample was 6.0 ± 4.0 cm. Most patients have progressed to advanced CRC (T1: T2: T3 = 0:2:18), and some patients have already developed regional lymph node metastasis (N0: N1: N2 = 8:9:3). The proportion of patients with lymph vascular invasion (present: absent = 5:15) and perineural invasion (present: absent = 2:18) indicates tumor progression, and most patients were treated (treated: untreated = 19:1). Detailed information on the clinical follow‐up is summarized in Table 1. Representative images of CD4+T/CD8+T cells and PD‐L1^+^/PD‐L1^-^ cases of CRC are shown in Figure 1.

Figure 1Immunohistochemistry of CD4+T cell, CD8+T cell, and PD‐L1 expression in CRC patients. (a) Immunohistochemistry of PD‐L1 in CRC. Representative images of (a–c). PD‐L1 positive in tumor cell samples (*n* = 19) and interstitial cell samples (*n* = 23). (d–f) PD‐L1 negative in tumor cell samples (*n* = 39) and interstitial cell samples (*n* = 14). (b) Immunohistochemistry of CD4 and CD8 in CRC patients. (a) Representative images of CD4‐positive cases (*n* = 57) are shown (100× magnification and 200× magnification). (b) Representative images of CD8‐positive cases (*n* = 53) are shown (100× magnification and 200× magnification).

**Figure:**
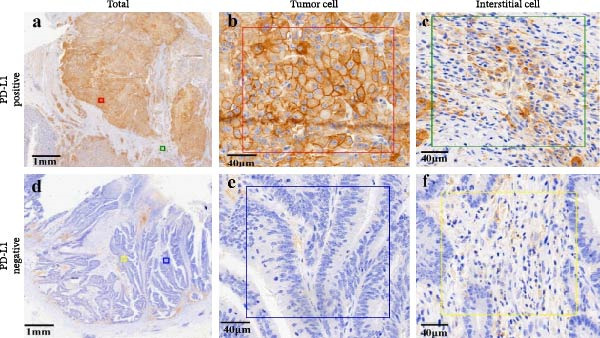
(a)

**Figure:**
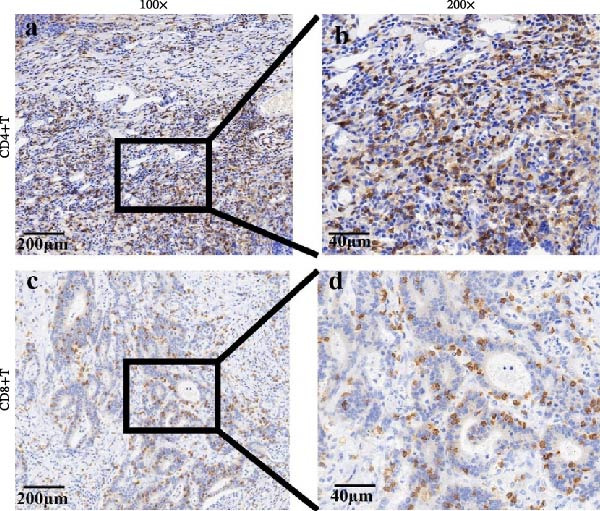
(b)

**Table 1:** Patient demographics.

	Total patients (*n* = 20)	
	*n*	%
Median age (range), years	62 (27–86)	
Sex		
Male	8	40
Female	12	60
Smoking		
Yes	3	15
No	17	85
Alcohol		
Yes	1	5
No	19	95
Pathologic stage		
Tumor (T)/nodes (N) stage		
T1	0	0
T2	2	10
T3	18	90
N0	8	40
N1	9	45
N2	3	15
Location of tumor		
Colon	8	40
Rectum	12	60
Tumor size, cm		
<5.0	9	45
≥5.0	11	55
Lymph vascular invasion		
Present	5	25
Absent	15	75
Perineural invasion		
Present	2	10
Absent	18	90
Treatment		
Yes	19	95
No	1	5

### 3.2. Correlation Between PD‐L1 and Immune Cell Infiltration in CRC

We fully utilized the TISIDB database to analyze the relationship between PD‐L1 and CD4+T/CD8+T cells in colorectal adenocarcinoma. PD‐L1 expression was significantly positively correlated with CD4+ and CD8+T cell infiltration (*p* < 0.05) (Figure 2a).

Figure 2Bioinformatics analysis of the correlation between PD‐L1 and immune cell infiltration in CRC. (a) The correlation of PD‐L1 with immune infiltrating in solid tumors. (b) PD‐L1 is associated with tumor purity and the degree of CD4+T/CD8+T cell immune infiltration in colon cancer (COAD) and rectal cancer (READ).

**Figure:**
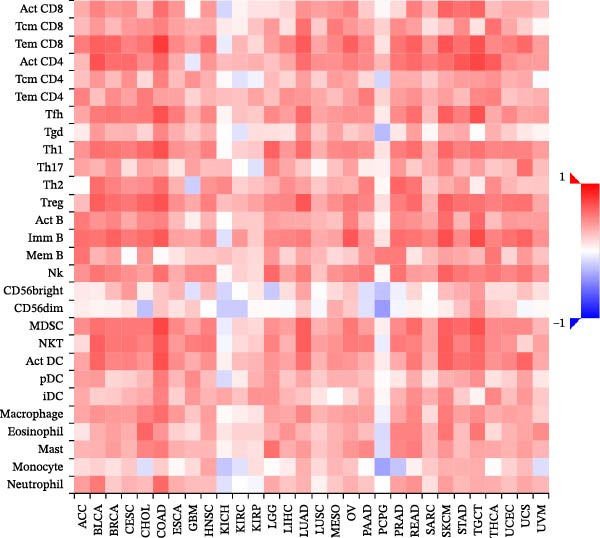
(a)

**Figure:**
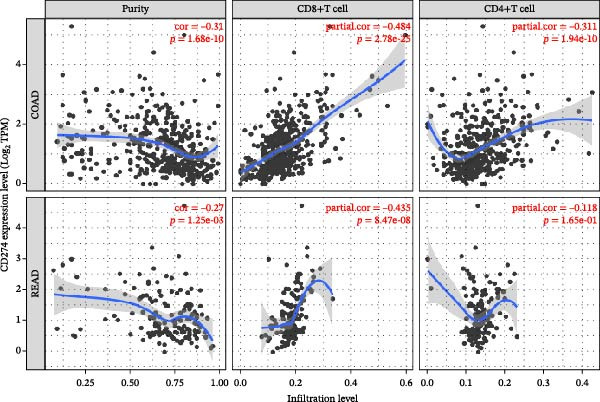
(b)

We further analyzed the correlation between PD‐L1 expression and immune cell infiltration in the TIME of colon and rectal cancers in the TIMER database. The expression level of PD‐L1 in colon cancer was negatively correlated with tumor purity (*r* = −0.31 and *p* = 1.68E‐10), CD8+T cell infiltration (*r* = 0.484 and *p* = 2.78E‐25), and CD4+T cell infiltration (*r* = 0.311 and *p* = 1.94E‐10). The gene expression level of PD‐L1 in rectal cancer was negatively correlated with tumor purity (*r* = −0.27 and *p* = 1.25E‐3) and positively correlated with CD8+T cell infiltration (*r* = 0.435 and *p* = 8.47E‐8) and CD4+T cell infiltration (*r* = 0.118 and *p* = 1.65E‐1) (Figure 2b).

### 3.3. Analysis of Differences and Correlation in TILs and PD‐L1 Expression Between Biopsy and Resection Specimens

The TIME differences between resection and biopsy specimens were assessed by CD4+T, CD8+T cell infiltration, and PD‐L1 expression. No statistically significant difference was observed in CD4+T cell counts and PD‐L1 expression between resection and biopsy specimens, and no statistically significant difference was observed in PD‐L1 expression levels when separately quantified in TCs and interstitial cells (ICs) (*p* > 0.05). In contrast, CD8+T cell infiltration was significantly higher in resection specimens compared to biopsy specimens (*p* < 0.05) (Figure 3a). With a sample size of 20 per group and *α* = 0.05, our study had ~80% power to detect only large effect sizes (Cohen‘s *d* ≥ 0.92).

Figure 3Differences and correlation between TIME in endoscopic biopsy and resection specimens in CRC (*n* = 20 per group). (a) Differences of CD4+/CD8+T cells and PD‐L1 expression in tumor cells between endoscopic biopsy and resection specimens (CD4: *p* = 0.6667; CD8:*p* = 0.0206; PD‐L1: *p* = 0.5034) and PD‐L1 expression in TC and IC between endoscopic biopsy and resection specimens (TC: *p* = 0.1413; IC: *p* = 0.6808). Statistical comparisons were performed using the paired *t*‐test for the CD4 and CD8 groups, while the Wilcoxon matched‐pairs signed‐rank test was applied to analyze PD‐L1 expression. (b–c) Correlation curves of PD‐L1 expression between endoscopic biopsy and resection specimens in TC (*r* = 0.9966 and *p* < 0.0001) and IC (*r* = −0.2301 and *p* = 0.3291). (d) Correlation curves of PD‐L1 expression between TC and IC in biopsy specimens (*r* = 0.7171 and *p* = 0.0004). (e–f) Correlation curves of CD4+T cells (*r* = −0.1323 and *p* = 0.5781) and CD8+T cells (*r* = 0.3850 and *p* = 0.0099) infiltration between endoscopic biopsy and resection specimens. (g) Correlation curves between CD4+T cells and CD8+T cell infiltration in resection specimens (*r* = 0.6710 and *p* = 0.0012). Data were analyzed using Spearman correlation, and *p*‐values were adjusted using the Benjamini–Hochberg FDR correction.

**Figure:**
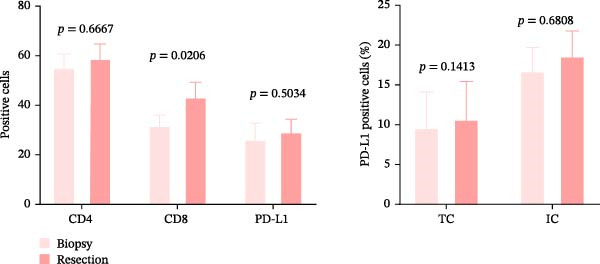
(a)

**Figure:**
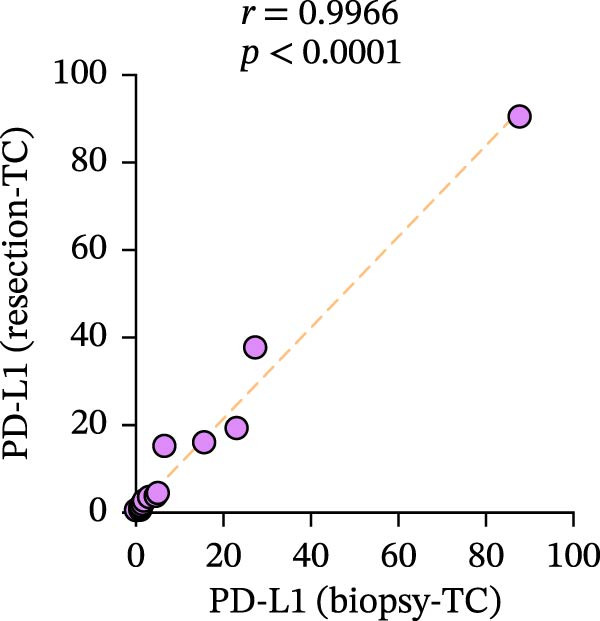
(b)

**Figure:**
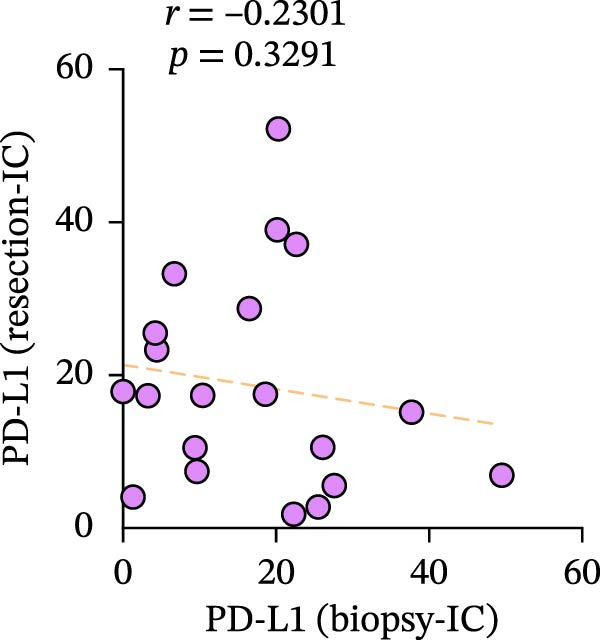
(c)

**Figure:**
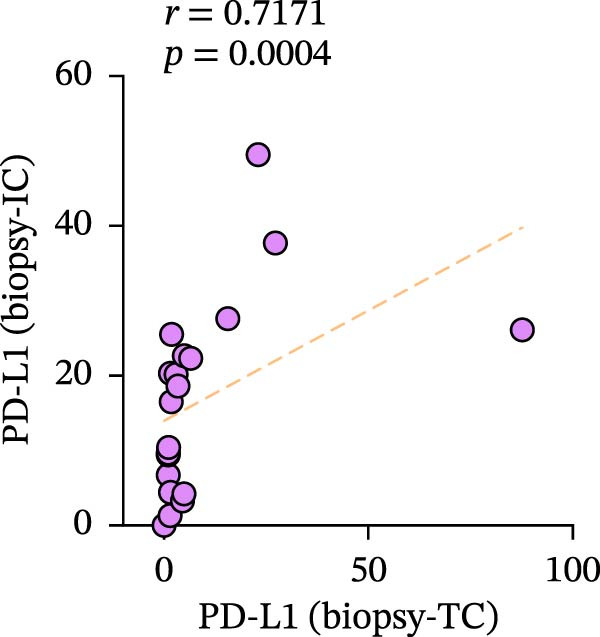
(d)

**Figure:**
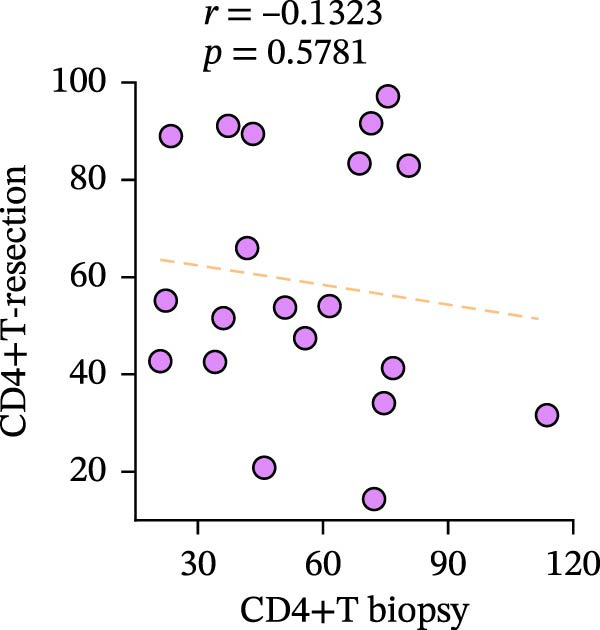
(e)

**Figure:**
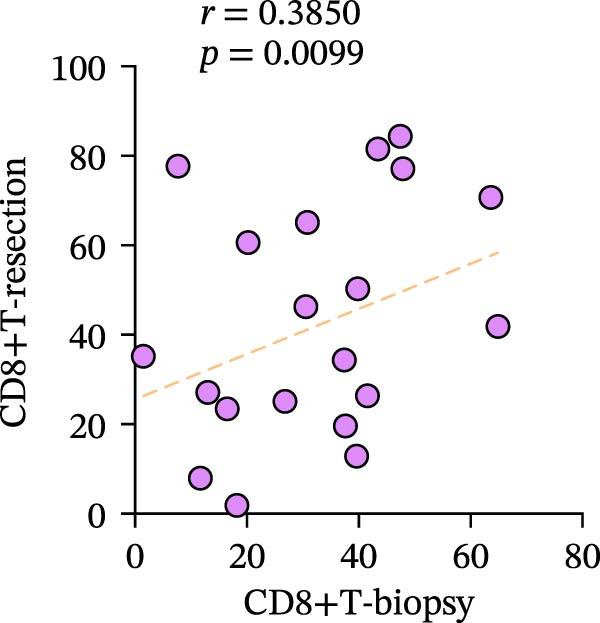
(f)

**Figure:**
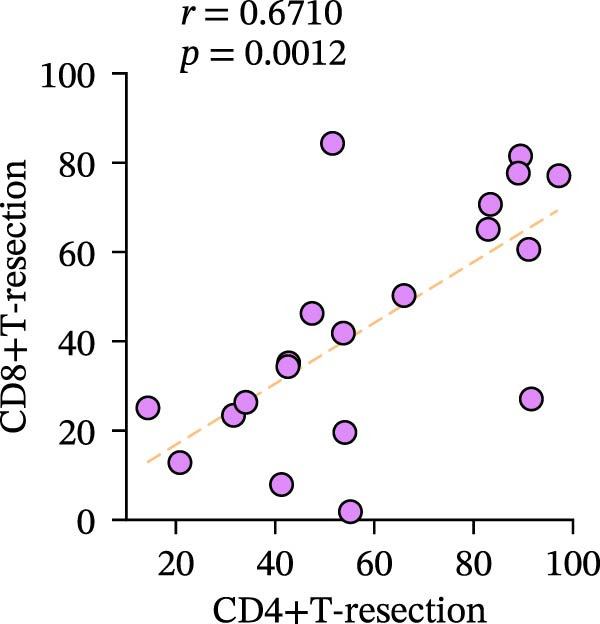
(g)

PD‐L1 expression in TC and IC samples in endoscopic biopsy was quantified in Figure 3d, and PD‐L1 expression showed increasing trends (*r* = 0.7171 and *p* = 0.0004), which also implied the consistency of PD‐L1 changes in the tumor and interstitium. Subsequently, we validated TC and IC PD‐L1 expression in surgical resection and endoscopic biopsy specimens, respectively. Notably, although PD‐L1 expression in TC showed positive correlation between two specimens (*r* = 0.9966 and *p* < 0.0001), we did not find an obvious relationship in its expression in the interstitium (*r* = −0.2301 and *p* = 0.3291); in fact, it is quite scattered and disordered (Figure 3b–d).

Subsequently, CD4+T/CD8+T cell infiltration was also detected and analyzed to determine their characteristics in different specimens. The consistency of CD4+T and CD8+T cell infiltration in resection specimens was firstly confirmed (*r* = 0.6710 and *p* = 0.0012) (Figure 3g), and the characteristics of the two specimens were further investigated. Surprisingly, the infiltration of CD8+T cells in the two specimens was positively correlated, as expected (*r* = 0.3850 and *p* = 0.0099), while the infiltration of CD4+T cells was not (*r* = −0.1323 and *p* = 0.5781) (Figure 3e, f).

### 3.4. Analysis of Differences and Correlation of Microenvironment in Primary and Metastatic Lesions of Metastatic CRC

To explore the immunological characteristics between metastatic and primary lesions in CRC, as well as their correlation and differences, TIME indicators of metastatic CRC were detected and analyzed in both metastatic and primary (as control) lesions.

A total of 29 cases were collected as metastatic CRC. The differential analysis indicates that both CD4+T (105.4 ± 20.82) and CD8+T cells (47 ± 9.418) in primary lesions are higher than in metastatic lesions of metastatic CRC (*p* < 0.05), while no statistically significant difference was observed in PD‐L1 expression (0.7586% ± 0.6212%) (*p*  > 0.05) (Figure 4a–c). Correlation analysis indicated that the number of PD‐L1 positive cells in primary lesions was positively correlated with metastatic lesions in metastatic CRC patients (*r* = 0.3826 and *p* = 0.0385) (Figure 4d). The same groups were used to compare the correlation between CD4+T and CD8+T cell infiltration. The number of CD4+T cells (*r* = 0.6931 and *p* < 0.0001) and CD8+T cells (*r* = 0.3803 and *p* = 0.0418) in primary and metastatic lesions were positively correlated (Figure 4e, f). Statistical power analysis demonstrated that an a priori power calculation confirmed >80% power to detect a medium effect (Cohen’s *d* ≥ 1.0; two‐sided *α* = 0.05). Statistical comparisons among the three groups were performed with Benjamini–Hochberg FDR correction.

Figure 4Differences and correlation between microenvironment in primary and metastatic lesions of metastatic CRC (*n* = 29 per group). (a–c) Differential analysis of (a) PD‐L1 (*p* = 0.4461 and FDR‐adjusted *p*‐value = 0.4461), (b) CD4+T cell (*p* = 0.0005 and FDR‐adjusted *p*‐value = 0.0015), and (c) CD8+T cell (*p* = 0.0136, FDR‐adjusted *p*‐value = 0.0204) as TIME indicators between primary and metastasis lesions in metastatic CRC. Statistical comparisons were performed using the paired *t*‐test, and *p*‐values were adjusted using the Benjamini–Hochberg FDR correction. (d–f) Correlation analysis of PD‐L1–positive cells and CD4+T/CD8+T cells as TIME indicators in primary and metastasic lesions in metastasic CRC. Correlations were assessed using Spearman’s rank correlation coefficient. The corresponding correlation coefficients (*r*) and *p*‐values are indicated in each panel.

**Figure:**
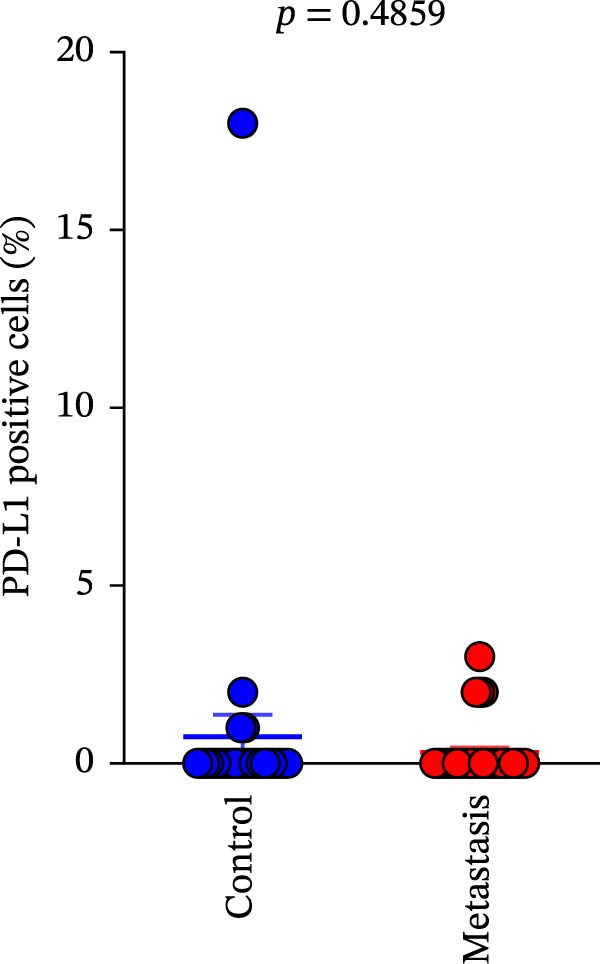
(a)

**Figure:**
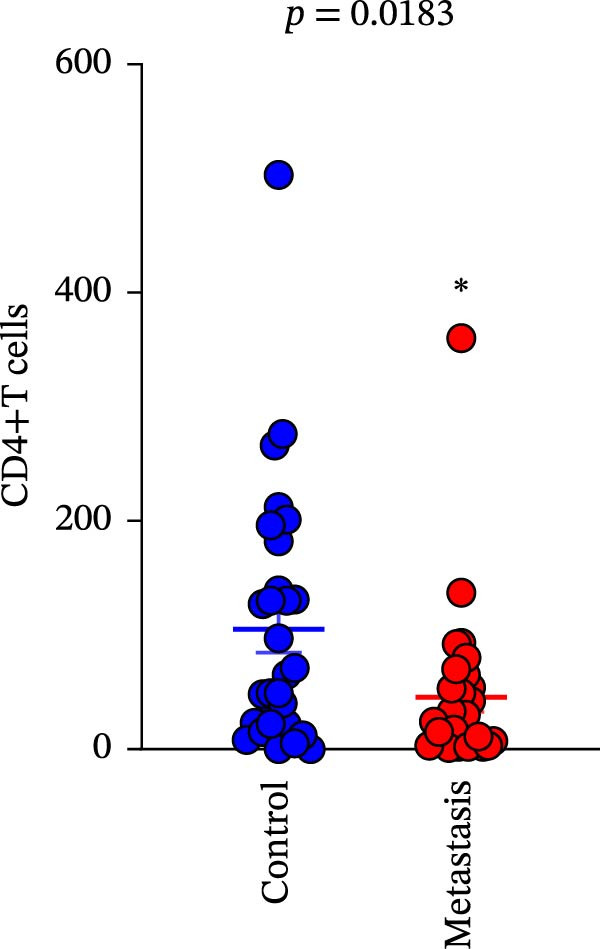
(b)

**Figure:**
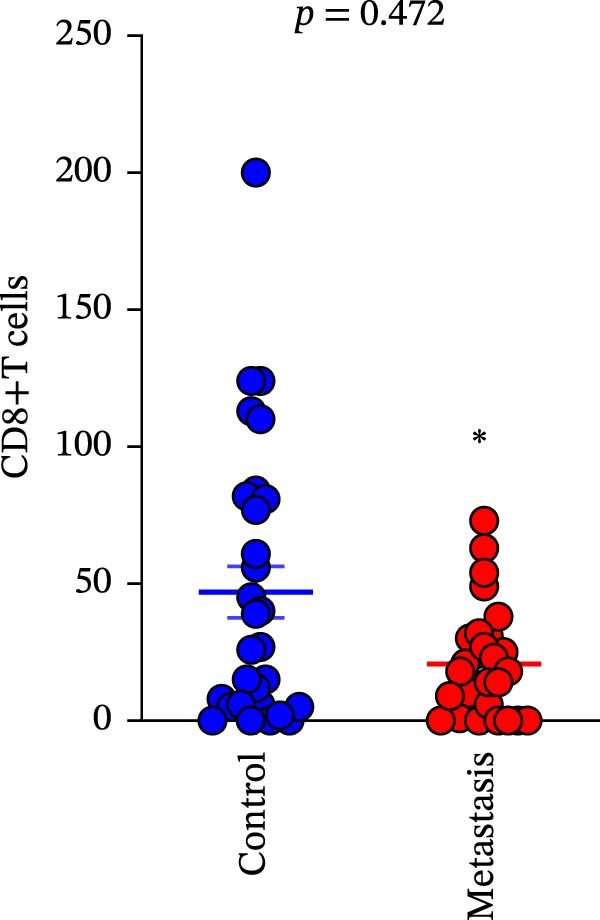
(c)

**Figure:**
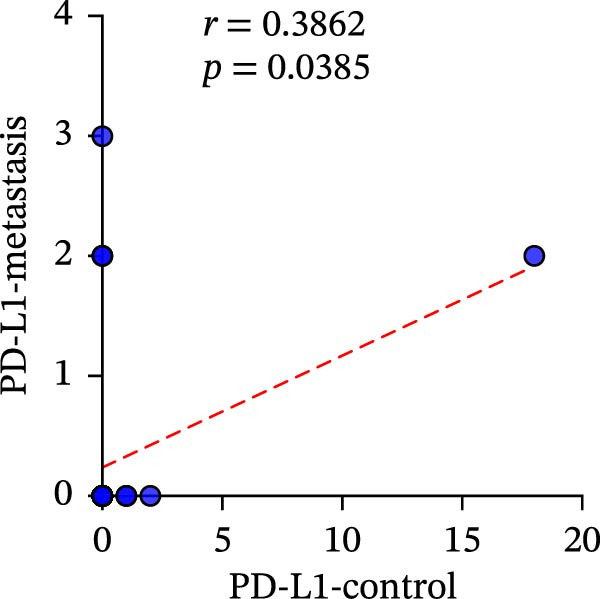
(d)

**Figure:**
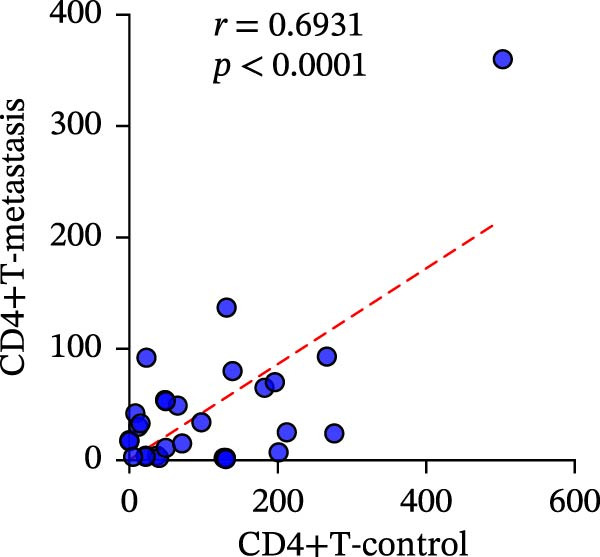
(e)

**Figure:**
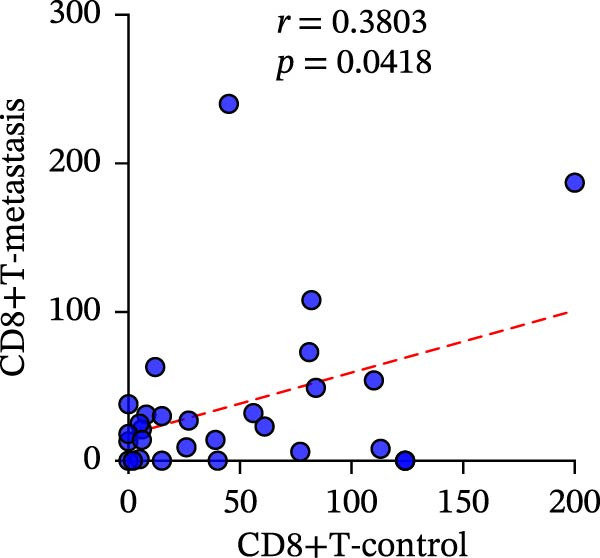
(f)

### 3.5. Differential Analysis of TILs and PD‐L1 Expression Between Primary Lesions in Metastatic and Nonmetastatic Groups

Comparison of primary lesions between nonmetastatic (*n* = 85) and metastatic (*n* = 29) CRC patients revealed that no significant differences were observed in PD‐L1–positive cells (metastatic samples: 5.012 ± 1.305%; nonmetastatic samples: 0.8148 ± 0.6667%) and CD4+T (metastatic samples: 91.18 ± 15.78; nonmetastatic samples: 133.2 ± 10.94)/CD8+T cell (metastatic samples: 47 ± 9.418; nonmetastatic samples: 52.16 ± 7.627) infiltrations between primary lesions of nonmetastatic patients and primary lesions of metastatic patients (all FDR‐adjusted *p* > 0.05) (Figure 5a–c).

Figure 5Immune‐related counts in primary lesions of nonmetastatic (*n* = 85) and metastatic (*n* = 29) CRC patients. (a) The number of PD‐L1–positive cells (*p* = 0.0770 and FDR‐adjusted *p*‐value = 0.1155). (b) The number of CD4+T cells (*p* = 0.0485 and FDR‐adjusted *p*‐value = 0.1155). (c) The number of CD8+T cells (*p* = 0.716 and FDR‐adjusted *p*‐value = 0.716). Statistical analysis was performed using independent samples *t*‐test, and *p*‐values were adjusted using the Benjamini–Hochberg FDR correction.

**Figure:**
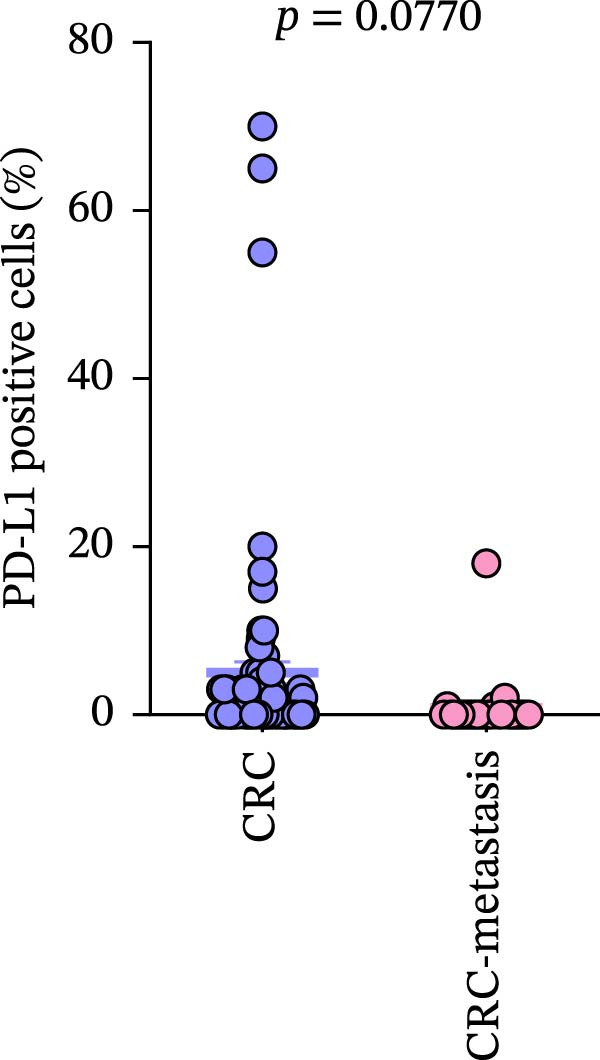
(a)

**Figure:**
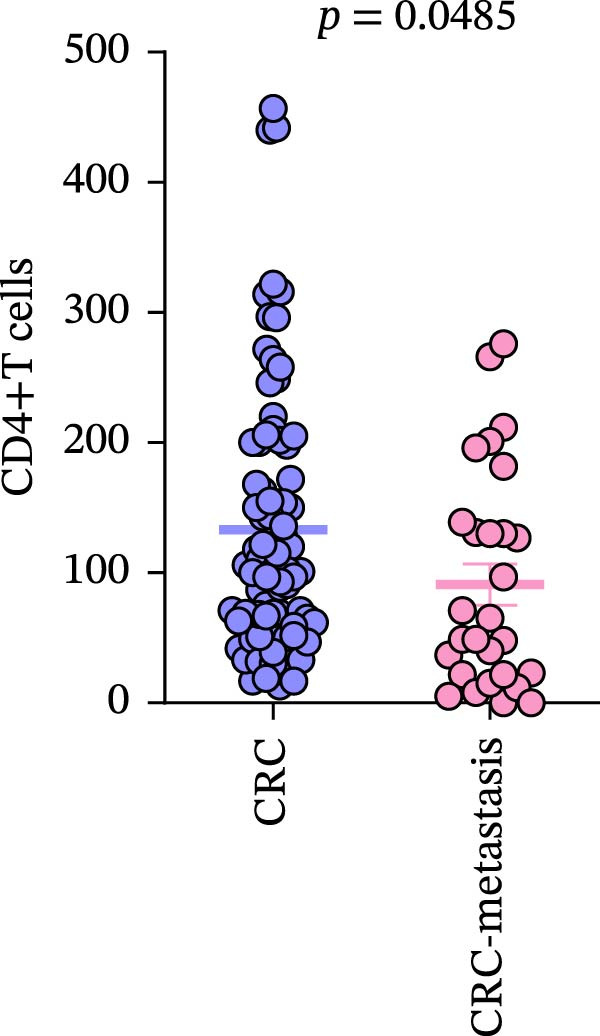
(b)

**Figure:**
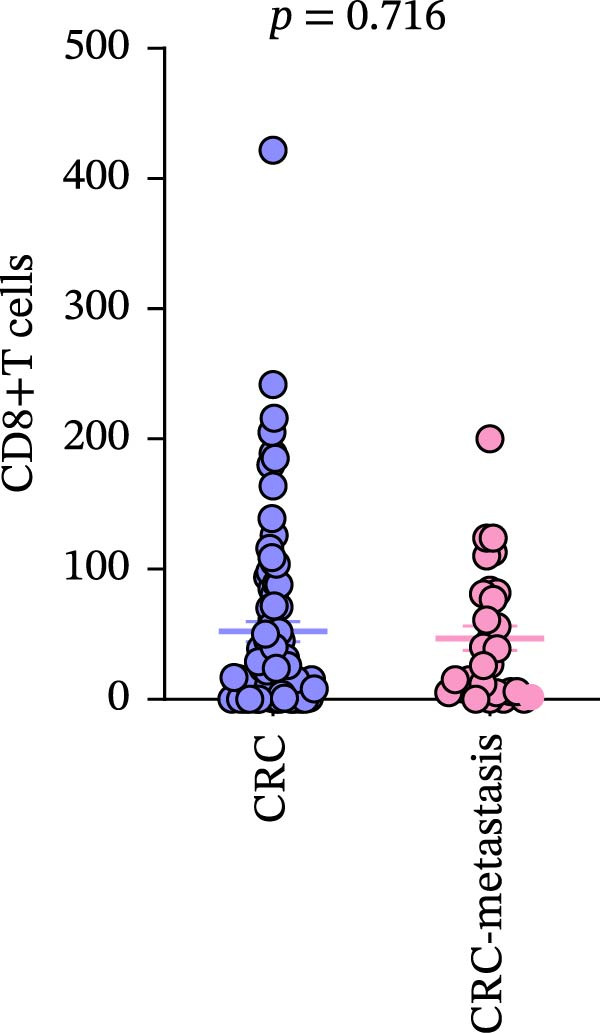
(c)

### 3.6. Correlation Analysis in TIME Components Between Primary and Metastatic Lesions

To clarify the quantitative relationship among the three TIME indicators in CRC, statistical analysis was performed in both primary and metastatic CRC lesions, the correlation between CD4+T and CD8+T cell infiltration itself led to the conclusion that, in primary cancer lesions, CD4+T and CD8+T cell infiltration was strongly positively correlated with PD‐L1 expression, and the presence was significantly correlated (CD4: *r* = 0.2949 and *p* = 0.0061; CD8: *r* = 0.4093 and *p* = 0.0001) (Figure 6a‐c). The metastatic lesions also showed a positive correlation between CD4+ and CD8+T cell infiltration and PD‐L1 expression (CD4: *r* = 0.5532 and *p* = 0.0019; CD8: *r* = 0.5999 and *p* = 0.0006). Meanwhile, consistency of CD4+ and CD8+T cell infiltration was observed in both primary (*r* = 0.510 and *p* < 0.0001) and metastatic lesions (*r* = 0.7363 and *p* < 0.0001), with statistical significance (Figure 6d, f).

Figure 6Correlation analysis of in TIL infiltration and PD‐L1 expression between primary tumor (*n* = 85) and metastatic tumor (*n* = 29). In primary lesions: (a) correlation between CD4+T cell and PD‐L1. (b) Correlation between CD8+T cell and PD‐L1. (c) Correlation between CD4+T/CD8+T cell infiltration. In metastasis: (d) correlation between CD4+T cell and PD‐L1. (e) Correlation between CD8+T cell and PD‐L1. (f) Correlation between CD4+T/CD8+T cell infiltration. Spearman’s correlation analysis was used to assess the correlations between CD8+T cell and PD‐L1, CD4+T cell and PD‐L1. Pearson’s correlation analysis was used to assess the correlations between CD4+T/CD8+T cell infiltration.

**Figure:**
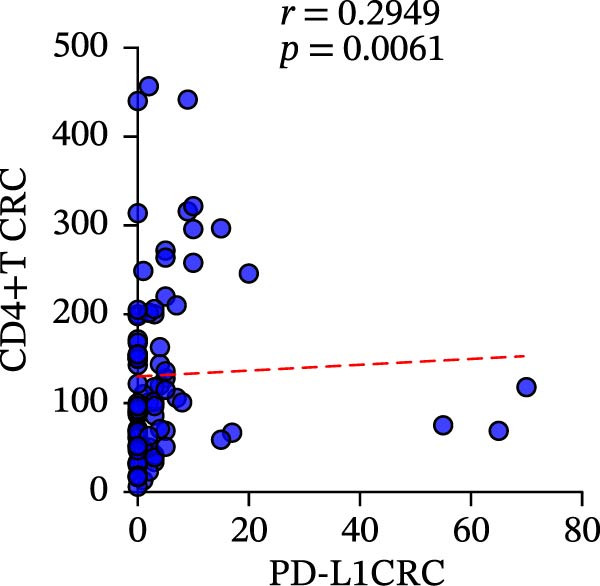
(a)

**Figure:**
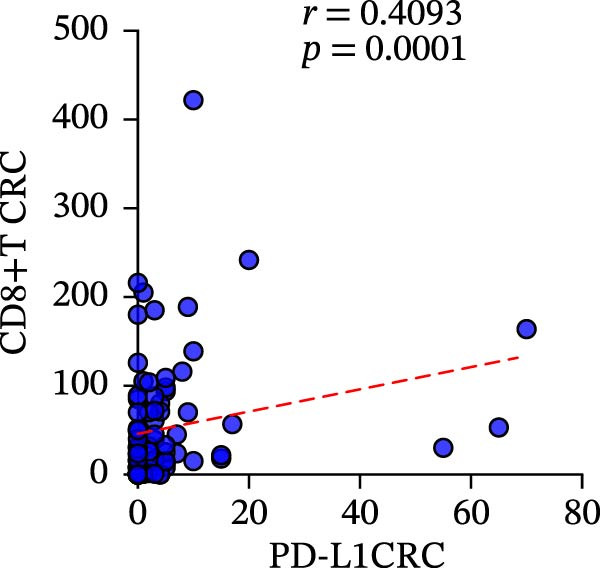
(b)

**Figure:**
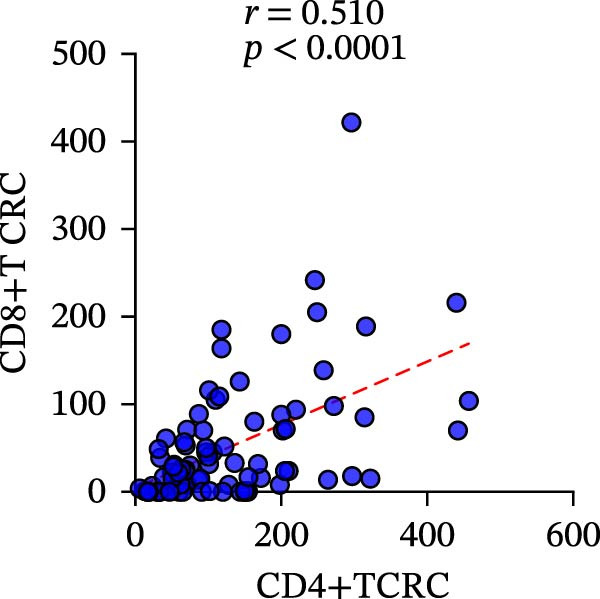
(c)

**Figure:**
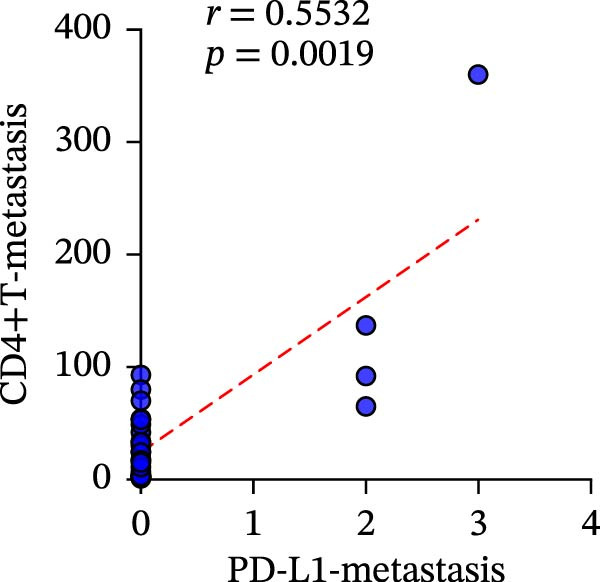
(d)

**Figure:**
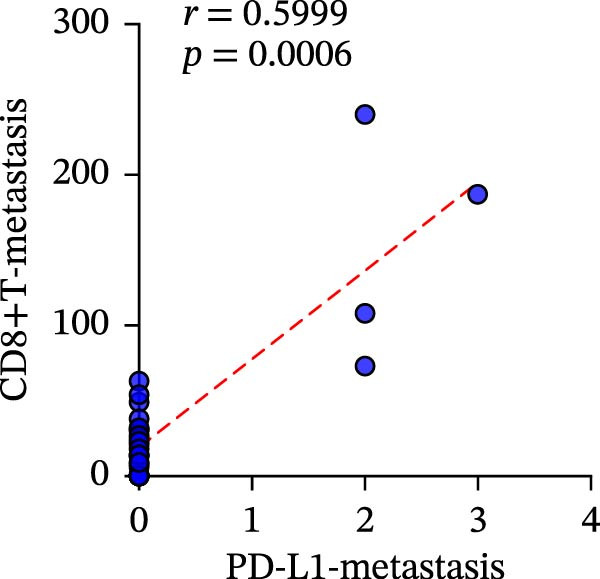
(e)

**Figure:**
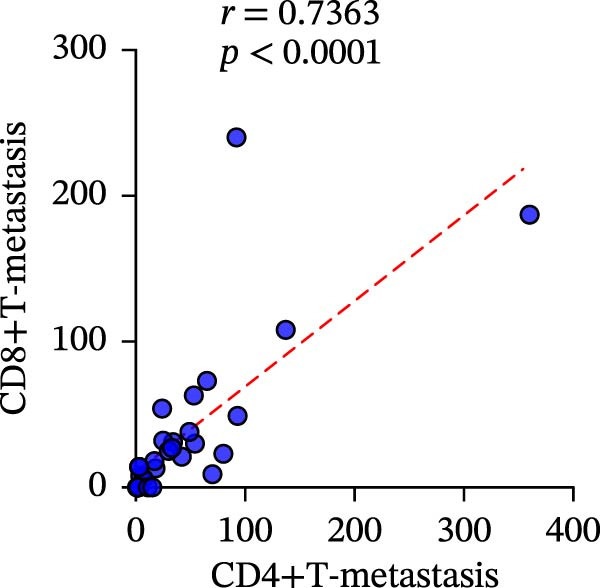
(f)

### 3.7. Analysis of PD‐L1 Distribution in High/Low CD4+T/CD8+T Cell Infiltration Group

We analyzed the positive expression of PD‐L1 in CRC with high and low CD4+T and CD8+T cell infiltration and the differential expression of PD‐L1 in CRC with MSS. As expected, PD‐L1 expression showed no obvious elevation in CRC with high CD4+T cell infiltration (5.405% ± 1.721%) compared to those with low CD4+T cell infiltration (4.628% ± 1.974%), *p* = 0.7679. The same counting was performed in CD8 group claiming more PD‐L1 expression in CRC with high CD8+T cell infiltration (7.93% ± 2.45%) than with low CD8+T cell infiltration (2.024% ± 0.5758%), *p* = 0.0227 (Figure 7a, b).

Figure 7(a–d) Comparison of immune markers between patient groups stratified by median infiltration levels of TILs (total *n* = 85; *n* = 42 for low group, *n* = 42 for high group; one case at the median was excluded). Quantification of PD‐L1–positive cells (%) in (a) high/low CD4+T cell infiltration (*p* = 0.7679). (b) High/low CD8+T cell infiltration (*p* = 0.0227). (c) Quantification of CD8+T cell in CD4 high/low group (*p* = 0.0002). (d) Quantification of CD4+T cell in CD8 high/low group (*p* = 0.0060). Statistical comparisons were performed using the independent samples *t*‐test. Differential analysis of TIME characteristics between CRC patients with MSI (*n* = 15) and MSS (*n* = 78). (e) Quantification of CD4+T cells (*p* = 0.0286 and FDR‐adjusted *p*‐value = 0.035). (f) Quantification of CD8+T cells (*p* < 0.001 and FDR‐adjusted *p*‐value = 0.0036). (g) Quantification of PD‐L1–positive cells (%) (*p* = 0.035 and FDR‐adjusted *p*‐value = 0.0035). (h) Quantification of Ki67‐positive cells (%) (*p* = 0.0031 and FDR‐adjusted *p*‐value = 0.0062). Statistical comparisons were performed using the independent samples *t*‐test, and *p*‐values were adjusted using the Benjamini–Hochberg FDR correction.

**Figure:**
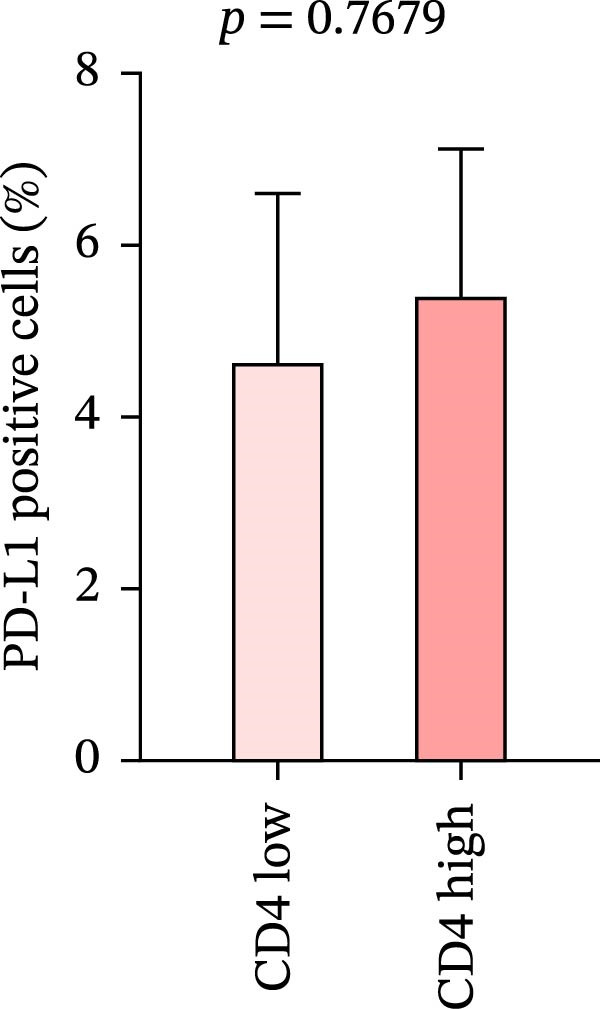
(a)

**Figure:**
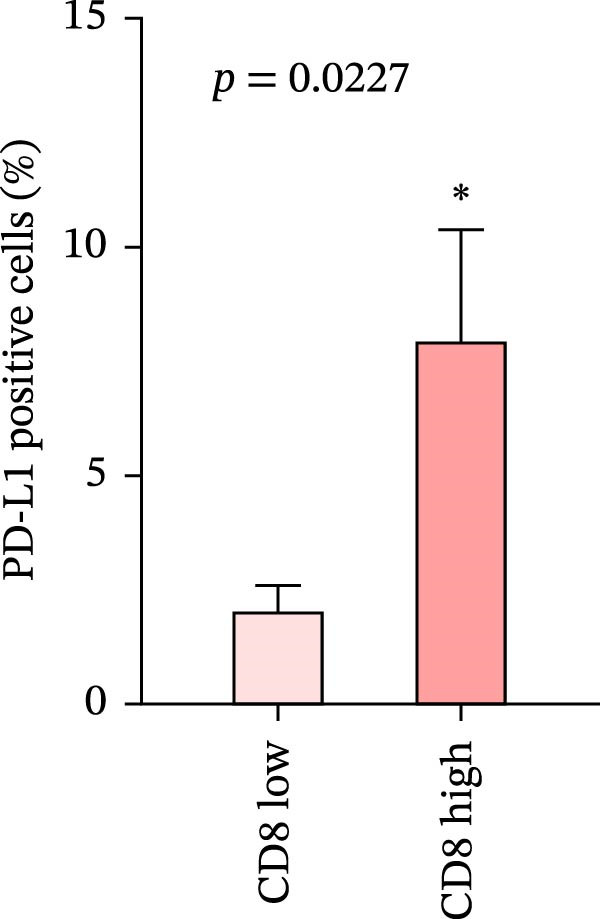
(b)

**Figure:**
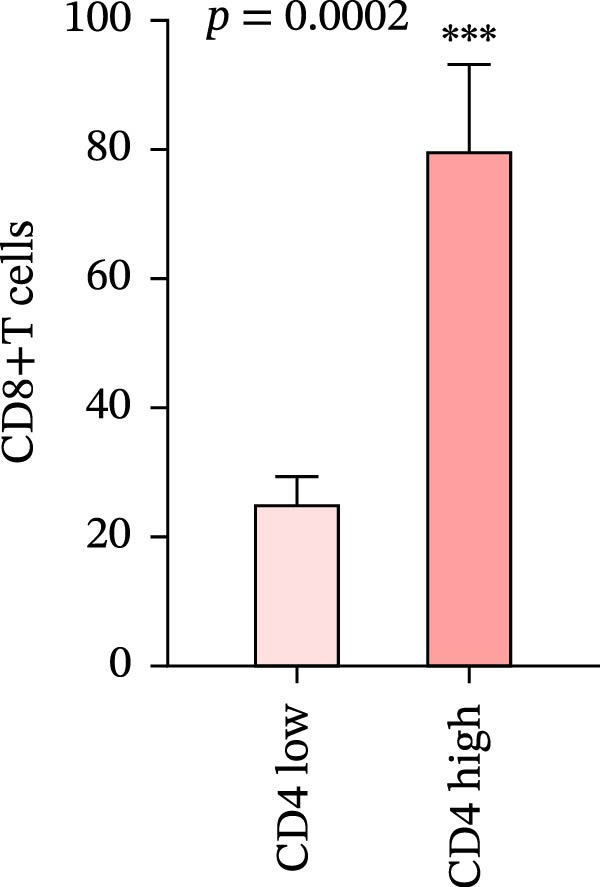
(c)

**Figure:**
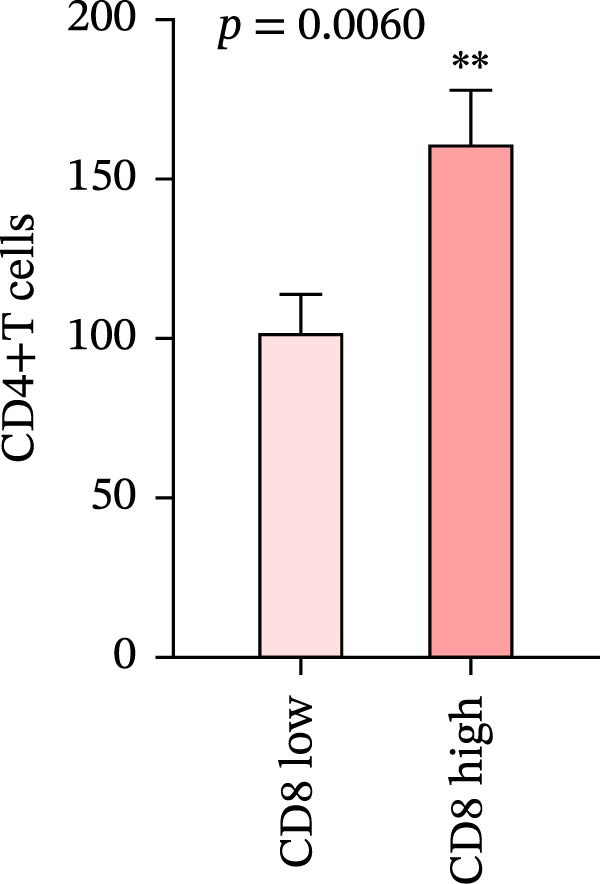
(d)

**Figure:**
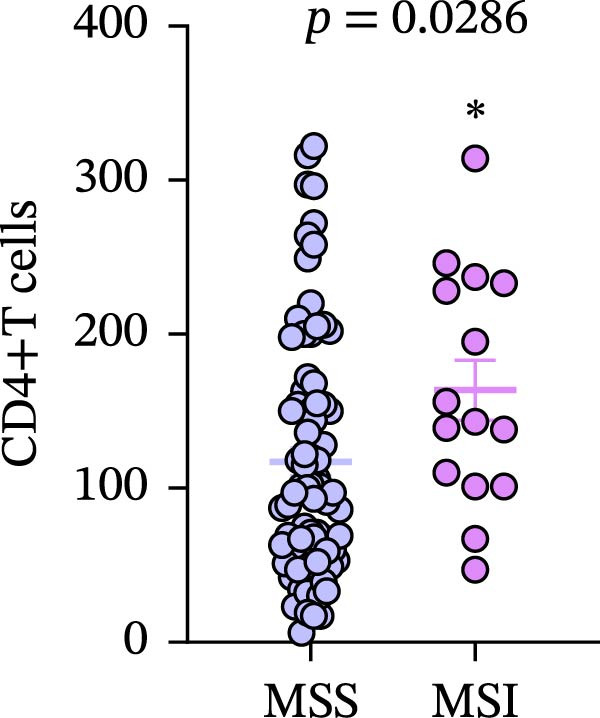
(e)

**Figure:**
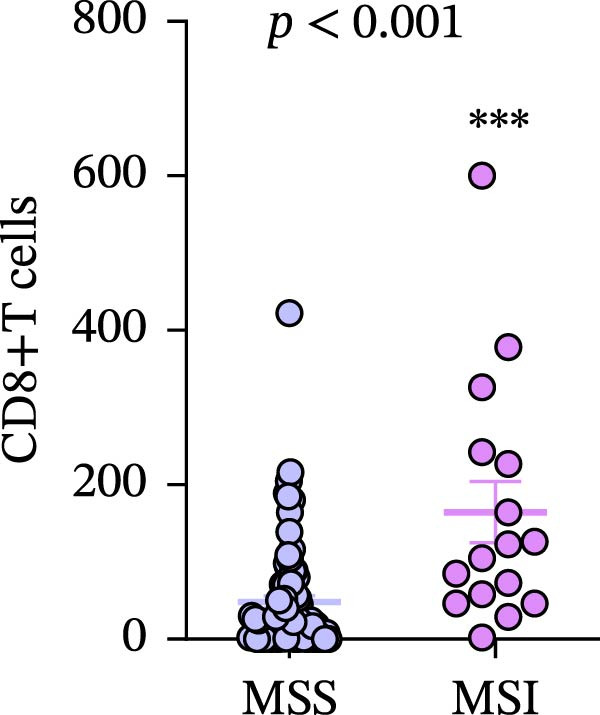
(f)

**Figure:**
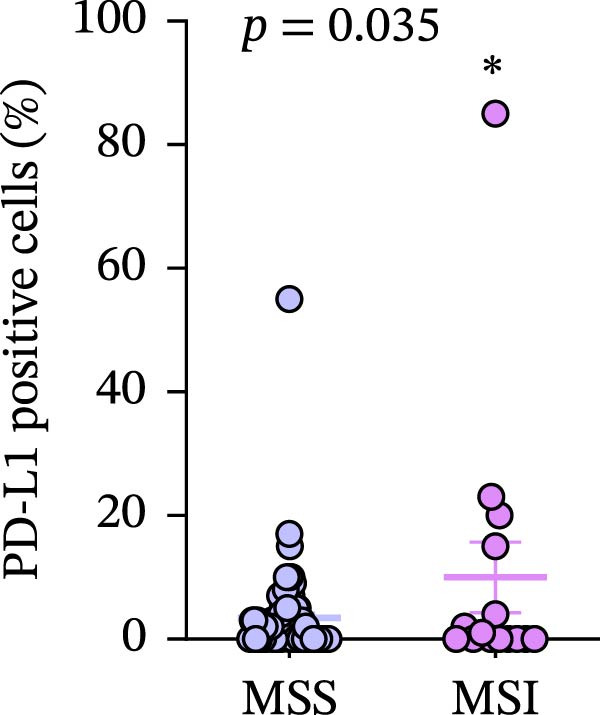
(g)

**Figure:**
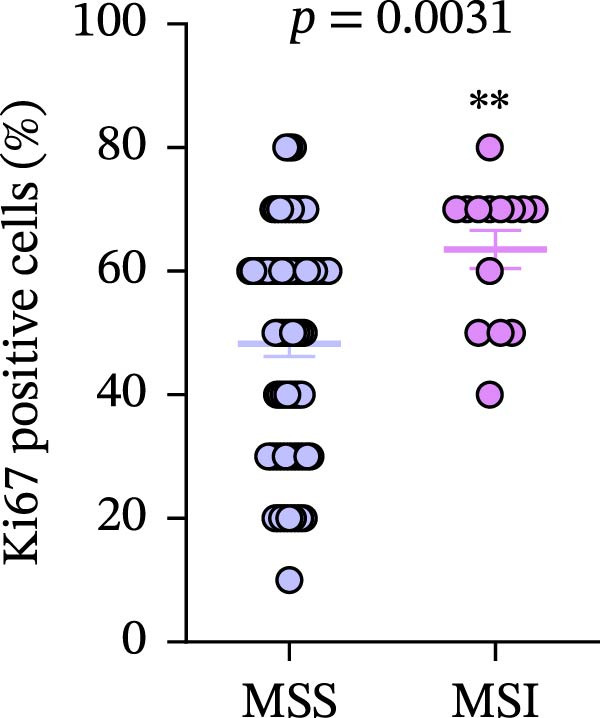
(h)

### 3.8. Differential Analysis of TIME Characteristics Between CRC Patients With MSI and MSS

No statistically significant difference in PD‐L1 expression was observed between CD4‐high and CD4‐low infiltration groups (*p* = 0.7679), whereas the CD8‐high infiltration group demonstrated significantly higher PD‐L1 expression compared to the CD8‐low infiltration group (*p* = 0.0227). Moreover, high infiltration levels of CD4+T cells were consistently associated with high infiltration levels of CD8+T cells, and vice versa (*p* < 0.05) (Figure 7a‐d). The counts of PD‐L1–positive cells and CD4+T/CD8+T cells were compared between CRC patients with MSI and MSS status, indicating higher average CD4+T cell counts in the MSI group (168.1 ± 20.42) than in the MSS group (117.2 ± 9.022), *p* = 0.0286 (Figure 7e). The average CD8+T cell count was also higher in the MSI group (172.3 ± 71.51) than in the MSS group (48.44 ± 7.612), *p* < 0.001 (Figure 7f). The PD‐L1–positive cell percentage was also higher in the MSI group (10 ± 5.732%) than in the MSS group (3.449% ± 0.7987%), *p* = 0.035 (Figure 7g). We also examined Ki67 expression to determine the degree of tumor malignancy. The MSI group had a higher percentage of Ki67‐positive cells (63.57 ± 3.075) than the MSS group (48.29 ± 2.072), *p* = 0.0031 (Figure 7h). Post hoc power analysis revealed that, at the *α* = 0.05 level, the current sample size provides approximately 60% statistical power to detect an effect size of *d* ≥ 1.0. Considering the relatively low prevalence of MSI subtype in the population (~15%), although this study is subject to statistical power limitations, we have included as many available samples as possible. All comparisons showed statistically significant differences after FDR correction.

## 4. Discussion

A growing body of studies has shown that the TIME plays an important role in the occurrence, development, invasion, and metastasis of CRC [34–36]. Among TIME, PD‐L1 is widely expressed in various TCs and is associated with immune evasion of TCs. Clinical scholars have pointed out that PD‐L1 plays a crucial role in the occurrence and development of various malignant tumors and can be used as diagnostic, prognostic, and/or predictive biomarkers for various types of cancer [37]. Meanwhile, CD4+T cells as well as CD8+T cell, as highly infiltrating lymphocytes in tumor tissues, play a vital role in the antitumor process in the body and are significantly infiltrated in CRC and its surrounding areas, which can initiate and regulate the body’s immune response [38–40]. However, since there are little researches on the differences in TIME in CRC endoscopic biopsy and resection specimens, as well as in metastatic specimens, this study investigated the degree of correlation among TIMEs as measured in biopsy, resection, and metastatic specimens in CRC patients.

A significant reduction in CD8+T cell infiltration was observed in biopsy specimens compared to surgical resection specimens (Figure 3). This specific difference likely points to a meaningful biological disparity between the two specimen types. However, caution is warranted in generalizing this finding, as similar significant differences were not seen for CD4+T cell density or PD‐L1 expression in TCs (Figure 3e‐g). The levels of PD‐L1 in various compartments are highly dynamic [41]. Some researchers claimed complete consistency of PD‐L1 expression in TC in primary cancer and lymph node metastatic cancer; however, expression consistency in IC was poor [42], while another study claimed significant differences [43]. Moreover, Evangelou et al. [44] found that, in breast cancer, PD‐L1 expression in TC and IC was associated with high TILs in the interstitium. Our results showed that PD‐L1 expression was consistent in both TC and IC in biopsy specimens, and a strong positive correlation in TC between biopsy and resection specimens was also observed (Figure 3a–d). However, our results showed no significant association between PD‐L1 expression in the interstitium of biopsy and resection (Figure 3c), the reasons for the differences between TC and IC are still worth discussing, and one possible explanation is the small sample sizes involved in our and other studies. In addition, there is a possibility that differences in study population, IHC assays, and antibodies used may all contribute to the controversial results observed. Future studies are, therefore, required to clarify it further.

This study systematically compares the TIME between primary and metastatic CRC lesions, revealing a significant positive correlation in immune features—particularly CD4+T cell infiltration. This finding suggests that when metastatic tissue is inaccessible, immune assessment of the primary tumor, especially CD4+T cell status, may provide complementary information for inferring the immune characteristics of metastases. Additionally, we confirmed a positive association between PD‐L1 expression and CD4+/CD8+T cell infiltration in both primary and metastatic lesions, consistent with prior reports. Notably, the stronger correlation between PD‐L1 positivity and high CD8+T cell infiltration further supports the role of CD8+T cell–driven adaptive immune resistance in CRC.

Our study, through differential analysis of the TIME in CRC patients, revealed significant differences in immune characteristics between MSI and MSS subtypes. Firstly, regarding the infiltration of key immune cells, we observed that MSI tumors exhibit a more active cellular immune state (Figure 7e, f). This finding aligns with the existing understanding that MSI CRC, due to its high mutational burden and generation of numerous neoantigens, is more readily recognized by the immune system, thereby recruiting and activating more effector T cells and creating an “immunologically inflamed” microenvironment [45]. This provides direct microenvironmental evidence for the typically better response of MSI CRC to ICI therapy. Secondly, corresponding to the enhanced immune infiltration, MSI tumors also demonstrated significant upregulation of PD‐L1 (Figure 7g). This is likely an adaptive feedback mechanism of TCs in response to intense T cell attack, known as “adaptive immune resistance.” This result further strengthens the theoretical rationale for applying PD‐1/PD‐L1 inhibitors in MSI CRC patients, indicating that this subtype not only exhibits immune cell infiltration but also pre‐existing expression of immune checkpoint molecules, making it more likely to benefit from blockade therapy. In addition, MSI CRC not only exhibited stronger immune cell infiltration (CD4+ and CD8+T cells) compared to MSS CRC but also showed a significantly higher TC proliferation index (Ki67) (Figure 7h). This reveals the dual characteristics of MSI tumors, which possess both high immunogenicity and high proliferative activity—likely two parallel manifestations resulting from the same core defect (deficiency in MMR function). However, it must be explicitly noted that the significant disparity in sample sizes between the MSI CRC (*n* = 15) and the MSS CRC (*n* = 78) in this study may introduce certain limitations. Although the key comparative results (such as CD4+ and CD8+T cell infiltration and PD‐L1 expression) demonstrated statistical significance, the imbalance in sample size, particularly the relatively small size of the MSI CRC (*n* = 15), may hinder a comprehensive assessment of intrasubtype heterogeneity within the MSI subtype and could potentially limit the precision of effect size estimation. While this sample distribution to some extent reflects the true epidemiological characteristics of MSI in the CRC population (accounting for ~15%), it may still reduce the statistical power of the study to detect small‐to‐moderate effect size differences. Therefore, future studies are needed to validate these associations in larger and more balanced cohorts and to further integrate multiomics data to more comprehensively elucidate the similarities and differences in the TIME between MSI and MSS subtypes, as well as their predictive value for treatment response.

With the deepening understanding of the tumor microenvironment, multiomics integration analysis and the role of specific molecular pathways in immune regulation have attracted increasing attention. For example, an analytical tool called Immuno‐oncology Biological Research has facilitated comprehensive analysis of TIME characteristics, immune interactions, and their impacts on immunotherapy outcomes, and systematic studies targeting the tumor microenvironment and antitumor immunity have revealed the prognostic value of various immune features [46]. Li et al. [47] identified, by leveraging single‐cell RNA sequencing and advanced computational tools, that the ratio of Dkk1^+^ (with immunosuppressive characteristics) to CALML5^+^ (associated with CD8+T cell infiltration) serves as a novel and robust biomarker for predicting immunotherapy response and patient prognosis, and the findings of this study emphasize the critical role of tumor heterogeneity and specific molecular regulation of TIME components, such as CD8+T cell infiltration, in head and neck squamous cell carcinoma. A growing body of evidence has shown that TIME exerts a significant influence on the occurrence, development, invasion, and metastasis of CRC. Regarding the specific molecular mechanism in regulating TIME in CRC, Syndecan‐2 has been confirmed to play an oncogenic role in CRC, which may be associated with its promotion of the formation of immunosuppressive “cold” tumors through the MAPK pathway [48, 49]. In addition, TIME also plays an important role in guiding clinical medication. Studies have reported that pharmacological interventions such as propranolol can inhibit CRC growth by activating autologous CD8+T cells and suppressing the tumor AKT/MAPK pathway [50], providing a pharmacological basis for understanding the regulatory mechanisms of immune cell function in the TIME. These studies all emphasize the importance of in‐depth analysis of microenvironmental heterogeneity in different tumor models. Based on this, we compared the differences in immune microenvironment indicators among different tissue specimens. Among TIME, PD‐L1 is widely expressed in various TCs and is associated with the immune evasion of TCs. Clinical scholars have pointed out that PD‐L1 can also promote the progression of various malignant tumors through immune escape; therefore, it can be used as a diagnostic and therapeutic marker [37]. Meanwhile, CD4+T/CD8+T cells, which are highly infiltrating lymphocytes, contribute to the antitumor process in the body and are significantly infiltrated in CRC and its surrounding areas, which can initiate and regulate the body’s immune response [38–40]. In addition to the several indicators we have studied, TIME heterogeneity is also reflected in other components, such as immune cells in innate immunity, whose molecular regulatory mechanisms have also attracted widespread attention. Studies have found that tumor‐derived exosomal miRNAs can activate the AKT signaling pathway in macrophages, driving tumor‐associated macrophages (TAMs) toward a protumor M2 polarization, thereby suppressing immune responses, promoting angiogenesis, and facilitating tumor growth [51, 52]. Additionally, the CD10+ neutrophil subset has been confirmed to directly interact with CD8+T cells via the SELPLG‐SELL ligand–receptor pair, driving their differentiation towards a terminally exhausted state. This leads to the loss of their antitumor function and subsequent tumor immune evasion [53]. Integrating these immune cell infiltration patterns with immune markers such as PD‐L1 expression, and further exploring the molecular mechanisms that regulate these indicators, enables a more comprehensive assessment of the dynamic changes in the TIME. This approach provides new insights into understanding the mechanisms underlying the formation of an immunosuppressive microenvironment and the mechanisms of primary resistance to immunotherapy. It is also expected to offer innovative theoretical foundations and potential therapeutic targets for precision immunotherapy strategies in CRC.

In conclusion, the TIME was assessed at three sites: CD4+T/CD8+T cell infiltration and PD‐L1 expression. We believe that these indicators in the TIME of resected specimens can be used to predict the progression of the tumor and the growth environment to some extent, facilitating clinical doctors to predict the treatment and medication in advance. However, ICs require further research because of their complexity. We believe that there is a large gap between it and primary CRC, especially in TIME and MSI. Future research on the treatment of CRC should focus more on new immunotherapy regimens and solve the problem of low immune invasion in metastatic CRC. Meanwhile, our study has some limitations. The sample size of biopsy and resection is comparatively small, and the number of metastatic specimens and CRC samples with MSS was insufficient. Future studies using a larger sample size are required to confirm the results observed in this study.

## Author Contributions

All authors have made substantial contributions to this work. Qi Liu did the formal analysis and methodology and wrote the original draft and review and editing. Wei Liu was in charge of data curation, resources, and software. Chunmei Zhang administrated the project, supervised the work, and acquired fundings. Yixuan Gao and Dongmei Feng investigated the data. Duo Deng was in charge of visualization. Yun Pan supervised the work.

## Funding

This research received funding from the Special Project for Training High‐level Health and Medical Technology Personnel in Yunnan Province (Grant H‐2024020) and the Yunnan Provincial Department of Science and Technology Basic Research Joint Special Project (Grant 202301A0070276).

## Ethics Statement

The study was approved by the Ethics Committee of the First Affiliated Hospital of Dali University (Approval Number DFY202050512001). All experiments were performed in accordance with the guidelines of the Declaration of Helsinki.

## Consent

All individuals participating in the study consented voluntarily to participate in the research and provided informed consent. All patients provided written informed consent from all subjects involved in the study.

## Conflicts of Interest

The authors declare no conflicts of interest.

## Data Availability

Publicly available datasets analyzed in this study were approved by the Ethics Committee of the First Affiliated Hospital of Dali University (Approval Number: DFY20240905001). These data are available at http://gepia2.cancer-pku.cn/ and http://timer.cistrome.org/.
